# College and Hospital Notes

**Published:** 1909-12

**Authors:** 


### COLLEGE AND HOSPITAL NOTES.

The Medical Department of the University of Buffalo has established an orthopedic clinic at the University Dispensary, 24 High Street, held daily from 4.00 to 5.00 p. m., with Dr. R. O. Meisenbach in charge. When needed, apparatus will be furnished at cost and, in especially worthy cases, will be given free of charge.

---

At the annual meeting of the Buffalo General Hospital held October 28, 1909, the following were reëlected officers for the ensuing year: president, Charles W. Pardee; vice-president, Henry M. Watson; treasurer, Elliott C. McDougal, and secretary, H. Edson Webster. There are twenty-seven trustees of the hospital and each year the terms of nine expire. These nine men and women were also reëlected: Henry M. Watson, Josiah Letchworth, Jacob F. Schoellkopf, Robert R. Hefford, George R. Howard, Spencer Kellogg, Mrs. Stephen M. Clement, Mrs. Bainbridge G. Folwell and Mrs. William Hamlin.

---

The American hospital, built and equipped through the generosity of the American colony in Paris, was formally opened, October 28, 1909, in the presence of American Ambassador White, M. Doumergue, Minister of Education, who represented the French government, members of the board of governors and the medical staff. The hospital is beautifully situated at Neuilly, where it is surrounded by spacious grounds. It contains twenty-five beds, many of which have already been endowed. Among those who have thus contributed to the permanency of the institution are J. P. Morgan, W. K. Vanderbilt and Miss Helen Gould.

---

Charles N. Crittenton, the New York millionaire, senior member of the wholesale drug firm, of Charles N. Crittenton & Co., widely known as the founder of the Florence Crittenton Rescue Homes for Girls, died at the Hotel Normandie, San Francisco, November 16, 1909, from pneumonia. He was ill less than a week. He was 76 years old. Mr. Crittenton founded seventy-three rescue homes in this country and several in Japan and China, which he named in memory of his daughter Florence, at whose dying request he entered the mission work. He was on a tour visiting the many rescue homes throughout the country, and arrived in San Francisco ten days before his death.

